# A new screening tool for SARS-CoV-2 infection based on self-reported patient clinical characteristics: the COV_19_-ID score

**DOI:** 10.1186/s12879-022-07164-1

**Published:** 2022-02-24

**Authors:** Pablo Diaz Badial, Hugo Bothorel, Omar Kherad, Philippe Dussoix, Faustine Tallonneau Bory, Majd Ramlawi

**Affiliations:** 1 Department of Emergency Medicine, La Tour Hospital, 1217 Geneva, Switzerland; 2 Research Department, La Tour Hospital, 1217 Geneva, Switzerland; 3 Department of Internal Medicine, La Tour Hospital and University of Geneva, 1217 Geneva, Switzerland

**Keywords:** COVID-19, SARS-CoV-2, Screening, Triage, Predictive score, Self-reported symptoms, Machine learning, Artifiicial Intelligence

## Abstract

**Background:**

While several studies aimed to identify risk factors for severe COVID-19 cases to better anticipate intensive care unit admissions, very few have been conducted on self-reported patient symptoms and characteristics, predictive of RT-PCR test positivity. We therefore aimed to identify those predictive factors and construct a predictive score for the screening of patients at admission.

**Methods:**

This was a monocentric retrospective analysis of clinical data from 9081 patients tested for SARS-CoV-2 infection from August 1 to November 30 2020. A multivariable logistic regression using least absolute shrinkage and selection operator (LASSO) was performed on a training dataset (60% of the data) to determine associations between self-reported patient characteristics and COVID-19 diagnosis. Regression coefficients were used to construct the Coronavirus 2019 Identification score (COV_19_-ID) and the optimal threshold calculated on the validation dataset (20%). Its predictive performance was finally evaluated on a test dataset (20%).

**Results:**

A total of 2084 (22.9%) patients were tested positive to SARS-CoV-2 infection. Using the LASSO model, COVID-19 was independently associated with loss of smell (Odds Ratio, 6.4), fever (OR, 2.7), history of contact with an infected person (OR, 1.7), loss of taste (OR, 1.5), muscle stiffness (OR, 1.5), cough (OR, 1.5), back pain (OR, 1.4), loss of appetite (OR, 1.3), as well as male sex (OR, 1.05). Conversely, COVID-19 was less likely associated with smoking (OR, 0.5), sore throat (OR, 0.9) and ear pain (OR, 0.9). All aforementioned variables were included in the COV_19_-ID score, which demonstrated on the test dataset an area under the receiver-operating characteristic curve of 82.9% (95% CI 80.6%–84.9%), and an accuracy of 74.2% (95% CI 74.1%–74.3%) with a high sensitivity (80.4%, 95% CI [80.3%–80.6%]) and specificity (72.2%, 95% CI [72.2%–72.4%]).

**Conclusions:**

The COV_19_-ID score could be useful in early triage of patients needing RT-PCR testing thus alleviating the burden on laboratories, emergency rooms, and wards.

**Supplementary Information:**

The online version contains supplementary material available at 10.1186/s12879-022-07164-1.

## Background

The current Coronavirus Disease 2019 (COVID-19) pandemic, represents one of the greatest medical challenges that the world had to face since decades. As COVID-19 quickly spread, various nonspecific clinical signs and symptoms have been reported making COVID-19 hard to differentiate from a broad range of respiratory tract infections [1, 2]. Diagnostic testing using real-time reverse transcription polymerase chain reaction (RT-PCR) has therefore been used to identify infected patients [3, 4] Several studies aimed to identify risk factors for severe acute respiratory syndrome coronavirus 2 (SARS-CoV-2) infection severity in order to anticipate intensive care unit (ICU) admissions [5–25].

However, few studies have been conducted on self-reported patient symptoms and characteristics, predictive of RT-PCR test positivity [4, 26–29]. Models involving loss of smell, loss of taste, cough and fever have been shown to reveal a higher infection likelihood [30, 31]. Although these predictive models were built on large cohorts, the proportion of infected patients was largely overestimated and symptoms may not have been collected with precision at the time of RT-PCR testing.

The main purpose of the present study was to identify predictive factors for SARS-CoV-2 infection based on self-reported patient symptoms and medical conditions, and construct a predictive score for patient screening at admission. Given the lack of availability of RT-PCR testing and delay in results, a reliable and quick tool may help clinicians on the front line in the prioritization for screening of patients at high risk for SARS-COV-2 infection.

## Methods

### Study design and participants

**Fig. 1:**
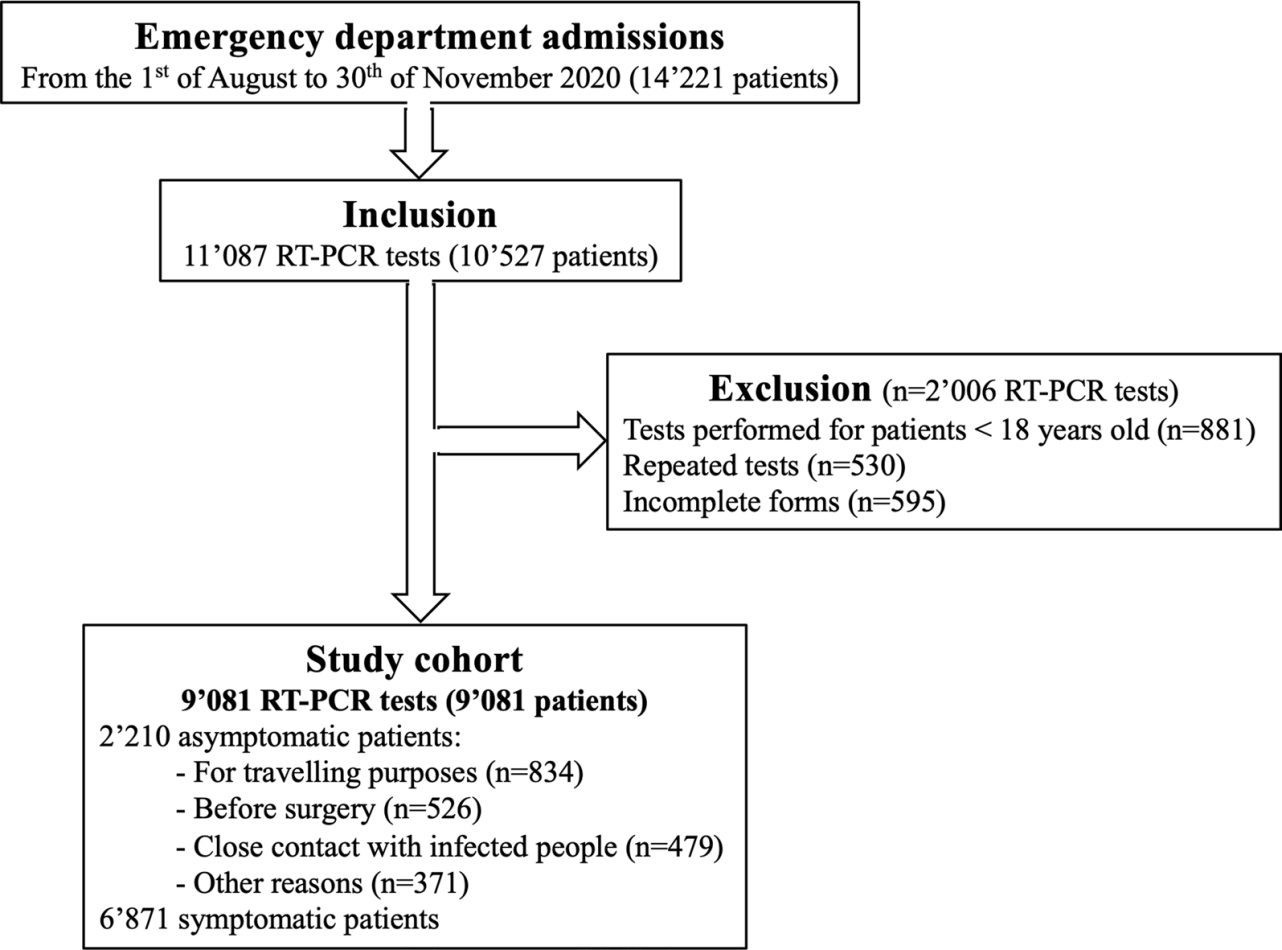
Study flowchart

A retrospective analysis of clinical data from 10,527 consecutive patients tested for SARS-CoV-2 infection was undertaken at the La Tour Hospital’s emergency center in Geneva (Switzerland) between the 1st of August and the 30th of November 2020. Our emergency department is an academically affiliated teaching center, requisitioned for SARS-COV-2 testing by the city’s health authorities. It represents the 2nd largest emergency in the city, accounting for 29,000 visits per year. All RT-PCR tests performed on patients younger than 18 years of age (n = 881, 7.9%) were excluded (Fig. 1). Since RT-PCR tests are associated with a variable false-negative rate [32], we excluded all non-final results from patients tested several times in our hospital due to worsening symptoms (n = 530, 4.8%). All incomplete forms were also excluded (n = 595, 5.4%). Ultimately, this led to the remainder of 9081 patients comprising 6871 symptomatic (75.7%) and 2210 asymptomatic (24.3%) cases with a unique final RT-PCR result for further analyses. Asymptomatic patients were tested for travelling purposes (n = 834, 9.2%), before surgery (n = 526, 5.8%), following a close contact with infected people (n = 479, 5.3%) or for other reasons (n = 371, 4.1%). This study was approved by the ethics committee of Geneva (CCER 2020-01742) and the need for informed written consent was waived owing to the urgent situation and the retrospective use of anonymized data.

### RT-PCR tests

SARS-CoV-2 infection was confirmed by positive RT-PCR tests on nasopharyngeal swab specimens. Specimens were sent to and analyzed by the National Reference Center for Emergency Viral Infections (CRIVE) at the Geneva University Hospital (HUG). PCR assays were performed using the Roche’s cobas® 6800 SARS-CoV-2 analyzer (Roche Molecular Systems, Branchburg, NJ) which received CE certification and the Emergency Use Authorization (EUA) by the U.S. Food and Drug Administration (FDA).

### Study variables

Each enlisted patient, filled a case report form (CRF) at the time of screening. The study variables included demographic data (age, gender, weight, height, profession) and a series of specific symptoms including cough, breathing difficulties, runny nose, sore throat, ear pain, headache, fever, muscle stiffness, back pain, diarrhea, nausea/vomiting, loss of appetite, loss of weight, loss of smell, loss of taste, dizziness, respiratory allergies and unusual fatigue. Other potential risk factors recorded included immunosuppression, diabetes, tobacco use, chronic pulmonary and heart disease, cancer as well as any history of close contact with people who have tested positive for SARS-CoV-2 infection. The data was then imported in a digital database, coded for anonymization, and stored on a secured hospital server.

### Statistical analyses

For baseline characteristics, continuous variables were reported as mean ± standard deviation with median and interquartile range (IQR), while categorical variables were reported as proportions. For non-Gaussian continuous data, differences between groups were evaluated using Wilcoxon rank-sum tests (Mann–Whitney U test), while for Gaussian continuous data, differences between groups were evaluated using unpaired Student t-tests. For categorical data, differences between groups were evaluated using the Fisher exact test. Univariable and multivariable logistic regressions were performed to determine associations between self-reported patient characteristics and COVID-19 diagnosis. Authors did not use imputation methods and performed their analyses on existing and complete data, thus the presented screening tool could only be used when information about all patient symptoms and characteristics is known. Sixty percent of the study population was randomly selected and contributed to build the multivariable logistic model (60%, training dataset), while the remaining part was kept to validate (20%, validation dataset) and test the model (20%, test dataset). The variables included in the shortened multivariable regression model were identified using the least absolute shrinkage and selection operator (LASSO) method. The regularization parameter used in this method was determined using a tenfold cross-validation, and set at one standard error from the λ that minimizes classification error (λ.1se). Collinearity was assessed using the Variance Inflation Factor (VIF) for each covariate, and was deemed acceptable if the maximum VIF did not exceed 2.0. Odds ratios (OR) and the 95% CI were calculated for each independent variable. Probability of being infected by SARS-CoV-2 was calculated as follows:

1 $${\text{Infection probability = }}\frac{1}{{1 + {\text{e}}^{{{ - }\left( {{\text{Intercept + }}\beta {\text{1X1 + }}\beta 2{\text{X2 + }} \ldots { + }\beta n{\text{Xn}}} \right)}} }}$$

With “Intercept” being the regression model intercept, and β the regression coefficient related to the independent variable X (X = 0 or 1). The regression coefficient for each independent variable selected in the multivariable model was multiplied by ten, rounded up to the nearest integer value, and used to build a predictive score: The Coronavirus 2019 Identification (COV_19_-ID) score. The regression coefficients were thereafter adjusted proportionally to set the maximum of the score at 100. The Receiver operating characteristic (ROC) curve was constructed and its area under the curve (AUC) evaluated. The optimal cutoff value was then calculated on the validation dataset to discriminate between infected patients and non-infected patients with the highest sensitivity and specificity (Youden Index). Two other thresholds were additionally described in a Additional file 1 to maximize either the sensitivity or the specificity of the COV_19_-ID score. To validate the variable selection in the LASSO regression, the AUCs obtained by the COV_19_-ID score and the entire multivariable model were evaluated and compared using a paired DeLong test. The sensitivity, specificity, positive and negative predictive values (PPV and NPV), positive and negative likelihood ratio (LR+ and LR−), F1 score and the Matthews correlation coefficient (MCC) were calculated on the test dataset based on the number of true positive (TP), false negative (FN), false positive (FP) and true negative (TN) cases. A bootstrap method with 1000 random resamples of the test dataset was used to calculate the 95% confidence interval (95%CI) of all aforementioned parameters. Statistical analyses were performed using R version 3.6.2 (R Foundation for Statistical Computing, Vienna, Austria). P-values < 0.05 were considered statistically significant.

## Results

A total of 14,221 patients were admitted to the emergency department from the 1st of August through the 30th of November 2020. 10,527 (74.0%) were tested for SARS-CoV-2 infection using RT-PCR tests, among whom 9081 were further analyzed (Fig. 1). The studied cohort included 4280 men (47%) with a mean age of 43.5 ± 15.6 years and a mean BMI of 25.1 ± 4.8 kg/m^2^. The most common reported symptoms were headache (38.6%), cough (38.4%), runny nose (34.0%), sore throat (31.4%), unusual fatigue (30.4%), muscle stiffness (27.3%) and back pain (22.4%) (Table 1).

**Table 1:** Patient characteristics for the entire cohort and by RT-PCR result subgroup (categorical data)

	Total (n = 9081)	Positive (n = 2084)	Negative (n = 6997)	*p-value*
	N (%)	N (%)	N (%)	
Symptomatic	6871 (75.7)	1993 (95.6)	4878 (69.7)	< *0.001*
Age (yrs)				0.185
18–39	3961 (43.6)	867 (41.6)	3094 (44.2)	
40–64	4226 (46.5)	1011 (48.5)	3215 (46.0)	
65–74	554 (6.1)	127 (6.1)	427 (6.1)	
≥ 75	340 (3.7)	79 (3.8)	261 (3.7)	
Male sex	4280 (47.1)	1049 (50.3)	3231 (46.2)	< *0.001*
Cough	3489 (38.4)	1105 (53.0)	2384 (34.1)	< *0.001*
Contact^a^ COVID-19+	3179 (35.0)	1004 (48.2)	2175 (31.1)	< *0.001*
Breathing difficulties	1034 (11.4)	281 (13.5)	753 (10.8)	*0.001*
Runny nose	3087 (34.0)	870 (41.7)	2217 (31.7)	< *0.001*
Sore throat	2850 (31.4)	629 (30.2)	2221 (31.7)	< *0.001*
Ear pain	546 (6.0)	123 (5.9%)	423 (6.0)	0.834
Headache	3502 (38.6)	1080 (51.8)	2422 (34.6)	< *0.001*
Fever	1246 (13.7)	584 (28.0)	662 (9.5)	< *0.001*
Diarrhea	1000 (11.0)	261 (12.5)	739 (10.6)	*0.013*
Nausea	787 (8.7)	210 (10.0)	577 (8.2)	*0.010*
Loss of smell	754 (8.3)	529 (25.4)	225 (3.1)	< *0.001*
Loss of taste	715 (7.9)	466 (22.4)	249 (3.6)	< *0.001*
Diabetes	289 (3.2)	76 (3.6)	213 (3.0)	0.177
Immunosuppression	92 (1.0)	15 (0.7)	77 (1.1)	0.136
Chronic pulmonary disease	149 (1.6)	32 (1.5)	117 (1.7)	0.768
Chronic heart disease	221 (2.4)	51 (2.4)	170 (2.4)	0.936
Cancer	226 (2.5)	46 (2.2)	180 (2.6)	0.379
Healthcare worker	409 (4.5)	93 (4.5)	316 (4.5)	0.952
Respiratory allergies	1121 (12.3)	247 (11.8)	874 (12.5)	0.448
Smoking	1408 (15.5)	208 (10.0)	1200 (17.2)	< *0.001*
Unusual fatigue	2762 (30.4)	845 (40.5)	1917 (27.4)	< *0.001*
Obesity (BMI > 30)	1212 (13.3)	286 (13.7)	926 (13.2)	0.557
Muscle stiffness	2481 (27.3)	936 (44.9)	1545 (22.1)	< *0.001*
Back pain	2031 (22.4)	784 (37.6)	1247 (17.8)	< *0.001*
Loss of appetite	930 (10.2)	410 (19.7)	520 (7.4)	< *0.001*
Loss of weight	160 (1.8)	78 (3.7)	82 (1.2)	< *0.001*
Dizziness	651 (7.2)	206 (9.9)	445 (6.4)	< *0.001*

Italic values indicate significant *p*-values (<0.05)

^a^Close contact with people who have tested positive for SARS-CoV-2 infection

### Predictive factors of SARS-CoV-2 infection

Among the tested population included in this study, 2084 patients (22.9%) were diagnosed with SARS-CoV-2 infection. Compared to patients with negative test results (n = 6997, 77.1%), confirmed cases were more likely of male sex (50.3% vs 46.2%) and symptomatic (95.6% vs 69.7%) (Table 1). The differences in terms of age (44.2 ± 15.6 vs. 43.3 ± 15.5 years, p < 0.001), BMI (25.4 ± 4.8 vs. 25.0 ± 4.8 kg/m^2^, p < 0.001), and time since symptoms onset (4.0 ± 6.0 vs 4.2 ± 5.0 days, p < 0.001) were statistically significant but not clinically relevant. Main symptoms reported by the infected group included loss of smell (25.4% vs 3.1%; p < 0.001), loss of taste (22.4% vs 3.6%; p < 0.001), fever (28.0% vs 9.5%; p < 0.001), muscle stiffness (44.9% vs 22.1%; p < 0.001), back pain (37.6% vs 17.8%; p < 0.001) and loss of appetite (19.7% vs 7.4%) (Table 1).

### Full multivariable model

SARS-CoV-2 infection was independently associated with loss of smell (OR, 9.4; 95% CI 6.9–12.8), fever (OR, 3.4; 95% CI, 2.8–4.1), increasing age (e.g. ≥ 75 y.o. vs. 18–39 y.o.: OR, 2.4; 95% CI 1.6–3.6), history of contact with an infected person (OR, 2.3; 95% CI 2.0–2.7), cough (OR, 2.1; 95% CI 1.8–2.5]), loss of taste (OR, 2.0; 95% CI 1.4–2.7), back pain (OR, 1.8; 95% CI 1.5–2.2), loss of appetite (OR, 1.8; 95% CI 1.4–2.3), muscle stiffness (OR, 1.7; 95% CI 1.5–2.1), as well as male sex (OR, 1.3; 95% CI 1.1–1.5) and headache (OR, 1.3; 95% CI 1.1–1.5). Conversely, COVID-19 was less likely associated with smoking (OR, 0.3; 95% CI 0.2–0.4), immunosuppression (OR, 0.3; 95% CI 0.1–0.7), ear pain (OR, 0.6; 95% CI 0.4–0.8), sore throat (OR, 0.7; 95% CI 0.6–0.9) and breathing difficulties (OR, 0.7; 95% CI 0.6–0.9) (Table 2).

**Table 2:** Uni- and Multivariable logistic regression of positive RT-PCR test (training data)

Variable	Univariable regression		Multivariable regression			
			Full model		LASSO model	
	OR (95% C.I.)	p-value	OR (95% C.I.)	p-value	Coeff	OR
Age group						
18–39	Ref.		Ref.			
40–64	1.1 (0.9–1.2)	0.440	1.3 (1.1–1.6)	< *0.001*		
65–74	1.1 (0.8–1.4)	0.719	1.7 (1.3–2.4)	< *0.001*		
≥ 75	1.3 (0.9–1.7)	0.175	2.4 (1.6–3.6)	< *0.001*		
Male sex	1.2 (1.1–1.4)	*0.006*	1.3 (1.1–1.5)	< *0.001*	0.035	1.05
Cough	2.2 (1.9–2.5)	< *0.001*	2.1 (1.8–2.5)	< *0.001*	0.437	1.5
Contact^a^ COVID-19 +	2.1 (1.8–2.3)	< *0.001*	2.3 (2.0–2.7)	< *0.001*	0.546	1.7
Breathing difficulties	1.3 (1.1–1.6)	*0.002*	0.7 (0.6–0.9)	*0.012*		
Runny nose	1.5 (1.3–1.7)	< *0.001*	1.1 (0.9–1.3)	0.175		
Sore throat	1.0 (0.8–1.1)	0.641	0.7 (0.6–0.8)	< *0.001*	−0.108	0.9
Ear pain	0.9 (0.7–1.2)	0.530	0.6 (0.4–0.8)	*0.002*	−0.098	0.9
Headache	2.0 (1.7–2.2)	< *0.001*	1.3 (1.1–1.5)	*0.003*		
Fever	3.8 (3.2–4.4)	< *0.001*	3.4 (2.8–4.1)	< *0.001*	1.004	2.7
Diarrhea	1.3 (1.0–1.5)	*0.021*	0.8 (0.7–1.1)	0.173		
Nausea	1.3 (1.0–1.6)	*0.031*	0.8 (0.6–1.0)	0.109		
Loss of smell	11.0 (8.9–13.6)	< *0.001*	9.4 (6.9–12.8)	< *0.001*	1.857	6.4
Loss of taste	7.5 (6.1–9.2)	< *0.001*	2.0 (1.4–2.7)	< *0.001*	0.432	1.5
Diabetes	1.3 (0.9–1.8)	0.144	1.2 (0.8–1.8)	0.312		
Immunosuppression	0.4 (0.2–0.9)	*0.045*	0.3 (0.1–0.7)	*0.010*		
Chronic pulmonary disease	0.9 (0.5–1.5)	0.754	0.7 (0.4–1.3)	0.322		
Chronic heart disease	0.8 (0.5–1.3)	0.415	0.6 (0.3–1.0)	0.055		
Cancer	0.8 (0.5–1.3)	0.380	0.9 (0.5–1.5)	0.665		
Healthcare worker	1.0 (0.7–1.3)	0.932	0.8 (0.6–1.2)	0.306		
Respiratory allergies	0.9 (0.7–1.1)	0.186	0.9 (0.7–1.1)	0.408		
Smoking	0.5 (0.4–0.6)	< *0.001*	0.3 (0.2–0.4)	< *0.001*	−0.672	0.5
Unusual fatigue	1.7 (1.5–1.9)	< *0.001*	0.9 (0.8–1.1)	0.237		
Obesity	1.1 (0.9–1.3)	0.368	1.0 (0.8–1.2)	0.651		
Muscle stiffness	2.6 (2.3–3.0)	< *0.001*	1.7 (1.5–2.1)	< *0.001*	0.390	1.5
Back pain	2.6 (2.2–2.9)	< *0.001*	1.8 (1.5–2.2)	< *0.001*	0.335	1.4
Loss of appetite	3.1 (2.6–3.7)	< *0.001*	1.8 (1.4–2.3)	< *0.001*	0.275	1.3
Loss of weight	2.9 (2.0–4.4)	< *0.001*	1.2 (0.7–1.9)	0.554		
Dizziness	1.7 (1.3–2.1)	< *0.001*	1.0 (0.8–1.4)	0.744		

Italic values indicate significant *p*-values (<0.05)

Multivariable model intercept: −2.153

OR, Odds ratio; CI, Confidence Interval; Coeff, coefficient

^a^Close contact with people who have tested positive for SARS-CoV-2 infection

### Creation and validation of the COV_*19*_-ID score

**Fig. 2:**
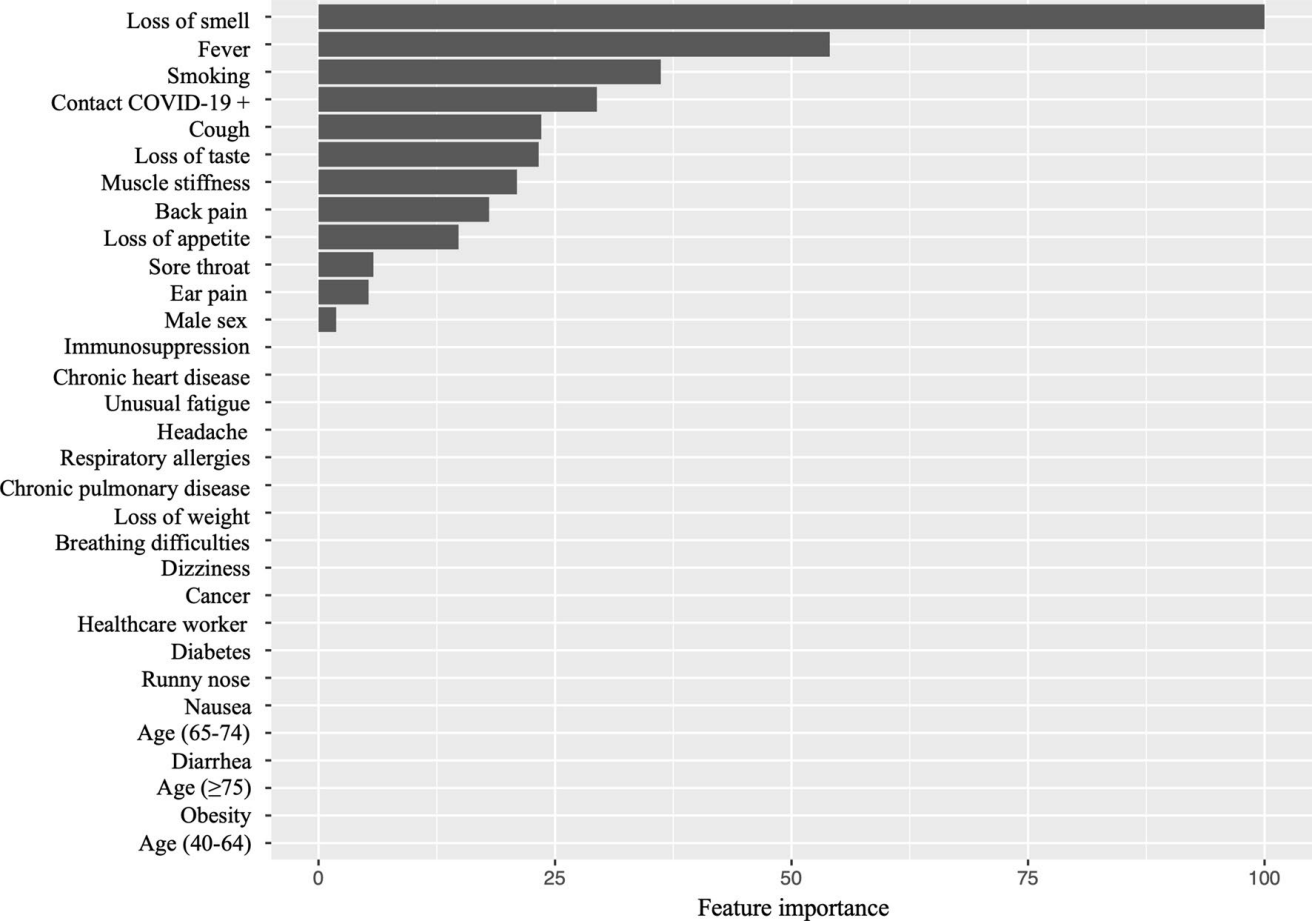
Feature importance determined by Least absolute shrinkage and selection operator (LASSO) regression

**Fig. 3:**
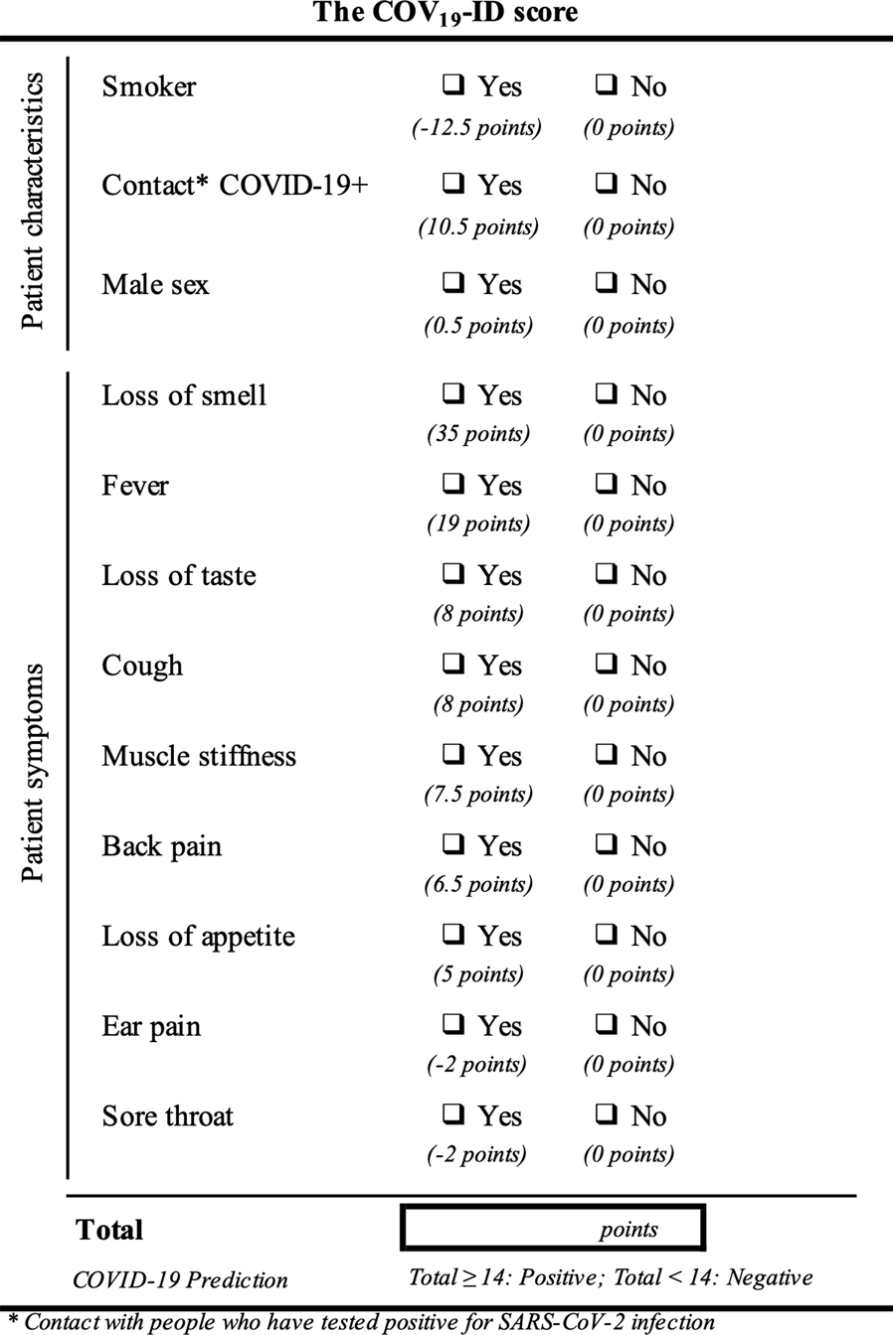
The COV_19_-ID score

Only twelve of the aforementioned predictors of SARS-CoV-2 infection were selected by the LASSO regression and used to create the COV_19_-ID score (Fig. 2 and 3). The COV_19_-ID score was thereafter calculated for all patients comprised in the validation dataset, which included 407 (22.5%) positive and 1399 (77.5%) negative cases.

**Fig. 4:**
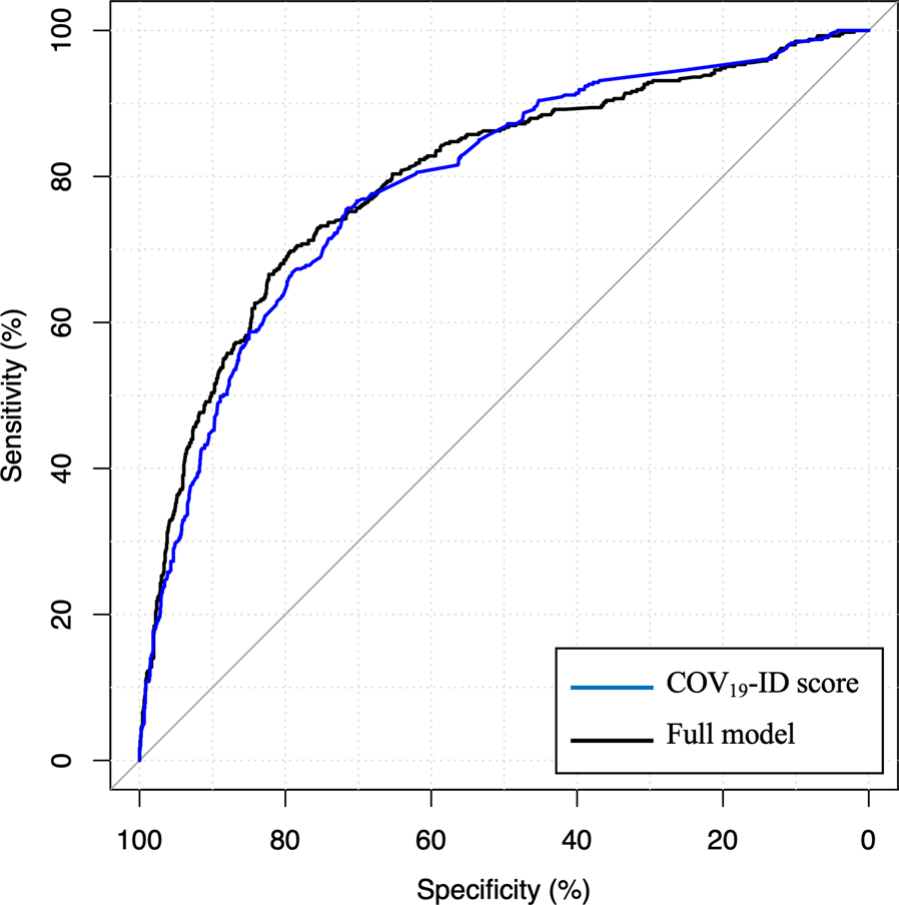
The Receiver-Operating Characteristic analysis for the COV19-ID score and the full multivariable model

On the validation dataset, the mean COV_19_-ID score was 10.0 ± 13.7 (median, 8.0; IQR, 0.0 –16.0) for patients with negative RT-PCR and 29.0 ± 20.9 (median, 25.0; IQR, 14.0–41.0) for patients with positive RT-PCR. The AUC obtained with the COV_19_-ID score was not significantly different from the AUC obtained with the full multivariable model (79.1% vs 79.8%, p = 0.121) (Fig. 4).

The COV_19_-ID score accuracy was 72.4% when maximizing both the sensitivity and specificity (cutoff value of ≥ 14 points). The sensitivity and specificity were 75.4% and 71.5% respectively, with a PPV of 43.5% and an NPV of 90.9% (Table 3). The F1 score and MCC were 0.55 and 0.40 respectively. Two other COV_19_-ID score thresholds were calculated to maximize either the sensitivity (≥ 8.5 points) or the specificity (≥ 25 points).

**Table 3:** Model performance on the validation and test datasets (maximizing sensitivity and specificity)

	Validation dataset (n = 1806)	Test dataset (n = 1815)	
		Actual	Bootstrap (95% CI)
True positive (TP)	307	345	
True negative (TN)	1000	1001	
False positive (FP)	399	385	
False negative (FN)	100	84	
AUC	79.1%	82.9%	(80.6%–84.9%)
Accuracy	72.4%	74.2%	(74.1%–74.3%)
Sensitivity	75.4%	80.4%	(80.4%–80.6%)
Specificity	71.5%	72.2%	(72.2%–72.3%)
Positive Predictive Value (PPV)	43.5%	47.3%	(47.2%–47.4%)
Negative Predictive Value (NPV)	90.9%	92.3%	(92.3%–92.4%)
Positive likelihood ratio (LR+)	2.64	2.90	(2.90–2.91)
Negative likelihood ratio (LR−)	0.34	0.27	(0.26–0.27)
F1 score	0.55	0.60	(0.59–0.60)
Mathews correlation coefficient (MCC)	0.40	0.46	(0.45–0.46)

### Test of the COV_19_-ID score

**Fig. 5:**
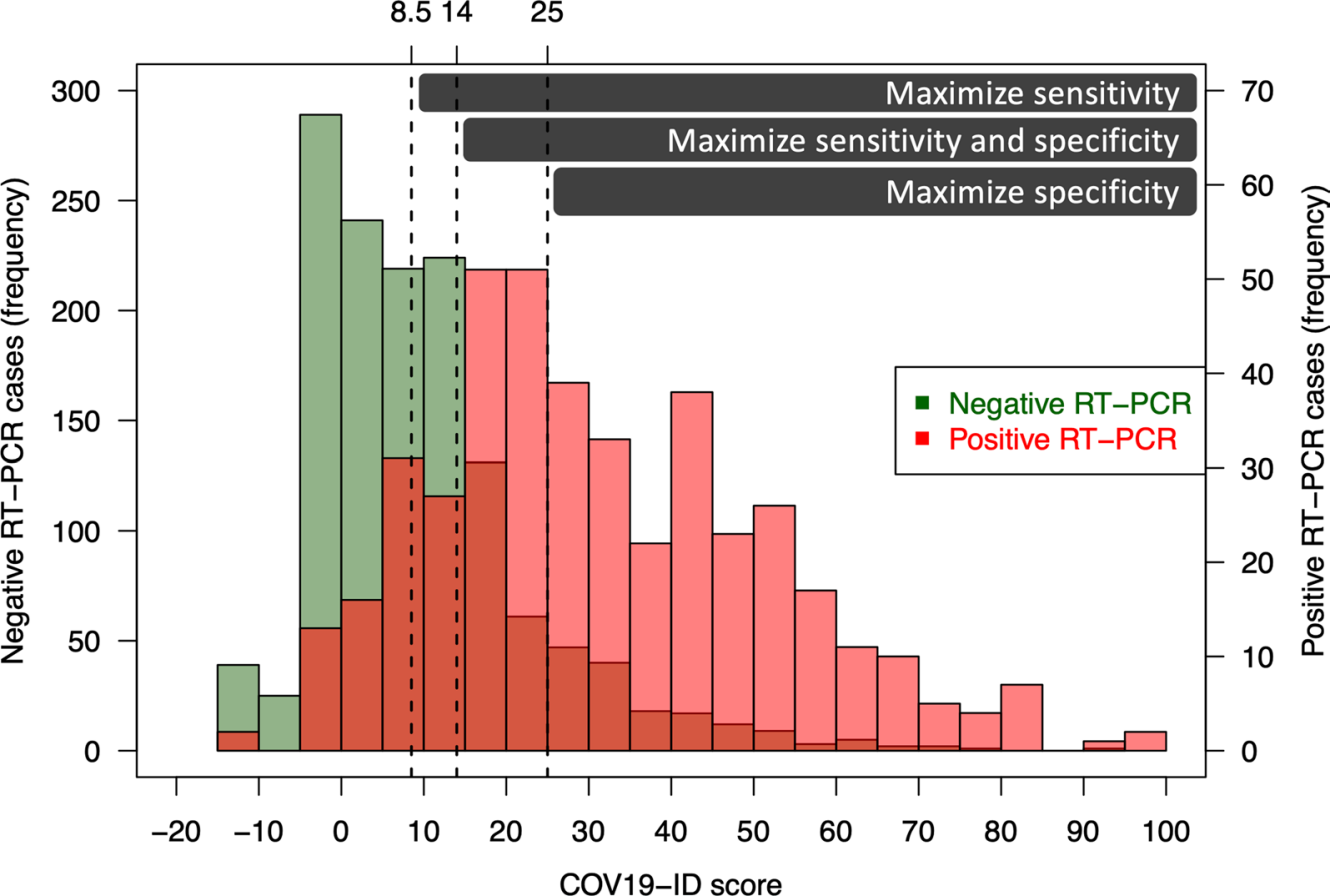
Histograms of COV_19_-ID score in negative and positive RT-PCR cases

Using the test dataset, which comprised 429 (23.6%) positive cases and 1386 (76.4%) negative cases, the AUC obtained with the COV_19_-ID score was 82.9% (95% CI 80.6%–84.9%). Using the cutoff value of ≥ 14 points, the accuracy was of 74.2% (95% CI 74.1%–74.3%) with a sensitivity and specificity of 80.4% (95% CI 80.4%–80.6%) and 72.2% (95% CI 72.2%–72.3%) respectively. The PPV was 47.3% (95% CI 47.2%–47.4%) and the NPV of 92.3% (95% CI 92.3%–92.4%) (Table 3). The F1 score and MCC were 0.60 (95% CI 0.59–0.60) and 0.46 (0.45–0.46) respectively. The comparison between the predicted probabilities of SARS-COV-2 infection and the RT-PCR test results is illustrated in Additional file 1. The model diagnostic performance using the three different thresholds is illustrated on Fig. 5 and detailed in Additional file 2.

## Discussion

The rapid spread of the COVID-19 pandemic and the need for mass testing invariably overwhelms laboratory capabilities resulting in increased result delays. To date, proposed screening tools [33, 34] mainly concern the detection of severe cases in order to anticipate for ICU admissions [5–25]. However, screening for SARS-CoV-2 infection at admission, may help discriminate between highly suspected patients needing quarantine measures or admission to COVID-19 dedicated units from those who could safely be discharged [35], while test results are pending. Our study presented and validated a new clinical tool (COV_19_-ID score) for SARS-CoV-2 infection based on the patient’s self-reported symptoms and medical history.

With an AUC of 83%, a sensitivity of 80% and a specificity of 72% for the prediction of SARS-CoV-2 infection in our test dataset, our screening tool compares well with the model of Menni et al. [31] who reported an AUC of 76% (sensitivity, 65%; specificity, 78%) in a United States cohort with a comparable proportion of infected patients (26% vs 24% in our test dataset). In their study, Zavascki et al. [36] created a score which included only 5 variables (patient age ≥ 60 years old, fever, dyspnea, coryza, and fatigue) that demonstrated an AUC of 88% in their validation dataset. It is worth noting however, that they did not use an external database for the validation process and that important symptoms such as loss of taste and loss of smell were not reported and incorporated in their model. In our study, 47% of the patients predicted of being infected by SARS-COV-2, truly had a positive test. This PPV is lower than that reported by Menni et al. [31] (69%). On the other hand, 92% of the patients predicted as not being infected had a negative test which is higher than that reported in the above study [31] (75%). These comparisons, however, should be interpreted with caution considering the differences in the studied population (e.g. the proportion of infected cases) and the cut-off value chosen for the prediction.

Main symptoms reported by COVID-19 patients included loss of smell, loss of taste, fever, muscle stiffness, back pain and loss of appetite. Known as a risk factor for transmission of the disease, exposure to a contagious person was only found in less than half of infected patients. This emphasizes the role of asymptomatic viral transmission in the population and the need for enhanced compliance with barrier measures. Although breathing difficulties has been largely described as one of the most prevalent symptoms associated with COVID-19 [37], our study revealed that in absence of cofounding factors, this symptom was rather suggestive of a non-SARS-CoV-2 infection. This finding was contradictory with those of Romero-Gameros et al. [38] but corroborated several recent studies that described a possible association between SARS-CoV-2 infection and lack of dyspnea (silent hypoxia) due to neurological damages [39, 40]. Another explanation would be that patients who did not present clinical signs suggestive of COVID-19, reported dyspnea because of other type of pneumonia or simply stress/anxiety before RT-PCR testing. Patients with sore throat and/or ear pain were also less likely to be infected by SARS-CoV-2, suggesting that these symptoms are more specific to other ears, nose and throat (ENT) diseases. Likewise, Spechbach et al. reported breathing difficulties and sore throat as predictors of a negative RT-PCR test [41]. Recent studies indicated that smokers tended to be less infected [4, 30, 42]. Our results corroborate these findings given that the odds of SARS-CoV-2 infection was two times less important for smokers.

Twelve variables were selected for the construct of the COV_19_-ID score owing to their high independent explanatory effect on SARS-CoV-2 infection. Among them, nine were potent risk factors for infection; comprising male sex, cough, loss of smell, loss of taste, fever, muscle stiffness, back pain, loss of appetite, and history of close contact with infected people. Our results are very similar to those published by Spechbach et al. who found that anosmia, fever, muscle pain, and cough were strong COVID-19 predictors [41]. In Menni et al.’s prediction model [31], loss of smell and taste, severe or persistent cough as well as loss of appetite were also highly predictive. Likewise, Apra et al. [30] reported that anosmic or ageusic patients were more likely to be infected but suggested to prioritize RT-PCR tests in patients with cough. Mao et al. [27] also found that exposure history was an independent risk factor for SARS-CoV-2 infection. Fever is usually one of the most reported symptoms in COVID-19 patients [37, 43]. In some studies, notably if performed in fever clinics [27], this symptom is so frequently reported (> 80%) in the global tested population that it does not help in the identification of COVID-19 patients. However, in a context of massive testing in a standard hospital, we showed that fever was reported by less than 20% of the symptomatic population. Our analyses revealed it to be the second most important factor associated with SARS-CoV-2 infection (behind loss of smell) at patient admission. It is worth noting that among all the aforementioned clinical signs, non-flu-like symptoms such as loss of smell or loss of taste are often considered in the screening process for SARS-CoV-2 infection owing to their greater specificity [44–46].

Although excluded variables from our model were not predictive factors of COVID-19, they could be of great interest in the prediction of infection severity and should still be considered during the medical encounter. For instance, the association between diabetes and the severity/mortality of patients with COVID-19 is well documented [47, 48] although this medical condition is not a risk factor per se for SARS-CoV-2 infection. Similarly, identified protective factors for SARS-CoV-2 infection might become a risk factor for COVID-19 severity. In our study, smoking was more likely to be considered as a protective factor for RT-PCR positivity, but it nonetheless contributes to COVID-19 severity once the patient is infected [49–52].

As to the use of this model in clinical practice, we suggest keeping the patients blinded to the score at the time of symptoms screening. Otherwise, patients might be tempted to report symptoms that are either strongly related or not to SARS-CoV-2 infection thereby reducing the diagnostic performance of the COV_19_-ID score. Furthermore, patients are unfamiliar to medical jargon and the medical lexicon used to describe the symptoms needs to be adapted to the population understanding for appropriate data collection (e.g. anosmia = loss of smell; ageusia = loss of taste, etc.). The strength of this score is its use at the time of admission. Solely based on patient-self reported information, it requires no health personnel assistance. Compared to models using laboratory and/or imaging data [53], this score is rapidly obtainable and does not require ancillary testing and/or patient radiation. Clinical uses of the COV_19_-ID score in a strained environment are large. Patients can be screened at admission and according to their score, directed to waiting areas planned for patients at low and high risk for SARS-CoV-2 infection thus preventing cross contamination [54]. Physicians or senior nurses can be appointed to patients at high risk areas thus optimizing resources. RT-PCR tests for patients at high risk could be prioritized to reduce result delays and the burden on laboratory facilities. Patients for whom a first test is negative but with a high COV_19_-ID score can be scheduled for a second test to decrease false negative results. For the same purpose, RT-PCR tests (gold standard) could also be used instead of rapid antigenic tests when patients present a COV_19_-ID score above a certain threshold (e.g. ≥ 25 points). Finally, a discriminating tool such as COV_19_-ID score has the potential to be incorporated in decision making algorithms used in telemedicine diagnostic strategies.

## Limitations

This retrospective study has several limitations. First, the number of patients with confirmed SARS-CoV-2 infection may be underestimated notably because of the suboptimal sensitivity of RT-PCR tests. To this date, the RT-PCR test remains the gold standard for SARS-COV-2 detection, although specimen sampling was refined and test turnaround times shortened. Although first repeated tests for patients with symptoms aggravation were excluded from the database, a number of patients with false negative results could still remain in the datasets thereby weakening the analyses. Second, a non-negligible rate of incomplete forms was excluded from our database (5%). However, the proportion of infected patients in the missing data was comparable to that of the studied dataset (21.5% vs 22.9%) and should therefore not represent an important bias. Our sample size may be criticized compared to multicentric or nationwide studies, however, we built our analysis on real data, gathered at the time of specimen collection, without using imputation methods for missing values. Third, the COV_19_-ID score was constructed from a local and homogeneous population and therefore needs to be validated prospectively in other populations. Furthermore, due to the retrospective nature of our study, we could not evaluate the diagnostic performance of the COV_19_-ID score on new COVID-19 variants (that may present non-classical symptoms) and on a vaccinated population. Fourth, since the statistical model used in this study did not include all patient symptoms and clinical characteristics, confounding effects that are unaccounted for could still be at play. Although we did not observe a relevant difference in terms of time since symptoms onset between infected and non-infected patients, such a factor should be further analyzed to reduce false negative results. Fifth, the COV_19_-ID score was established on data collected between August and November where COVID-19 was the predominant circulating virus. Because the seasonality has a considerable impact on the onset of viral diseases other than COVID-19; late spring, early summer and winter viruses such as the influenza virus may trigger flu like symptoms thus weakening the diagnostic performance of the COV_19_-ID score and increasing false positive rates (lower specificity). Further studies are therefore needed to estimate the impact of seasonality on the use of the COV_19_-ID score. Finally, the use of the COV_19_-ID score in a context of massive testing may be associated with a higher false negative rate at the time of RT-PCR testing (lower sensitivity) due to a higher proportion of infected patients that may not present the majority of COVID-19 predictive factors yet.

## Conclusions

This study presented and validated a new screening tool (the COV_19_-ID score) for SARS-CoV-2 infection detection based on patients self-reported symptoms and medical history. This score has an acceptable diagnostic performance and might be useful in early triage of patients needing RT-PCR testing thus hopefully alleviating the burden on laboratories, emergency rooms, and wards.

## Supplementary Information

**Additional file 1: **Predicted probabilities of SARS-COV-2 infection compared to RT-PCR test results.

**Additional file 2: **Model performance on the test dataset using three different thresholds to maximize either sensitivity, specificity, or both.

## Data Availability

The datasets used and/or analysed during the current study are available from the corresponding author on reasonable request.

## Abbreviations

SARS-CoV-2: Severe Acute Respiratory Syndrome Coronavirus 2
COV_19_-ID score: Coronavirus 2019 Identification score
OR: Odds ratio
95% CI: 95% Confidence Interval
TP: True positive
TN: True negative
FP: False positive
FN: False negative
PPV: Positive predictive value
NPV: Negative predictive value
LR+: Positive likelihood ratio
LR−: Negative likelihood ratio
MCC: Matthews correlation coefficient
RT-PCR: Real-time reverse transcription polymerase chain reaction
EUA: Emergency Use Authorization
FDA: Food and Drug Administration
CRF: Case report form
IQR: Interquartile range
VIF: Variance Inflation Factor
ROC: Receiver operating characteristic
AUC: Area under the curve
ENT: Ears Nose and Throat

## References

[1] Fu L, Wang B, Yuan T, Chen X, Ao Y, Fitzpatrick T (2020). Clinical characteristics of coronavirus disease 2019 (COVID-19) in China: a systematic review and meta-analysis. J Infect.

[2] Ferreira-Santos D, Maranhao P, Monteiro-Soares M (2020). Covidcids Identifying common baseline clinical features of COVID-19: a scoping review. BMJ Open.

[3] Corman VM, Landt O, Kaiser M, Molenkamp R, Meijer A, Chu DK (2020). Detection of 2019 novel coronavirus (2019-nCoV) by real-time RT-PCR. Euro Surveill.

[4] de Lusignan S, Dorward J, Correa A, Jones N, Akinyemi O, Amirthalingam G (2020). Risk factors for SARS-CoV-2 among patients in the Oxford Royal College of General Practitioners Research and Surveillance Centre primary care network: a cross-sectional study. Lancet Infect Dis.

[5] Bhargava A, Fukushima EA, Levine M, Zhao W, Tanveer F, Szpunar SM (2020). Predictors for severe COVID-19 infection. Clin Infect Dis.

[6] Chang MC, Park YK, Kim BO, Park D (2020). Risk factors for disease progression in COVID-19 patients. BMC Infect Dis.

[7] Chen R, Liang W, Jiang M, Guan W, Zhan C, Wang T (2020). Risk factors of fatal outcome in hospitalized subjects with coronavirus disease 2019 from a nationwide analysis in China. Chest.

[8] Flook M, Jackson C, Vasileiou E, Simpson CR, Muckian MD, Agrawal U (2021). Informing the public health response to COVID-19: a systematic review of risk factors for disease, severity, and mortality. BMC Infect Dis.

[9] Galloway JB, Norton S, Barker RD, Brookes A, Carey I, Clarke BD (2020). A clinical risk score to identify patients with COVID-19 at high risk of critical care admission or death: an observational cohort study. J Infect.

[10] Gao J, Huang X, Gu H, Lou L, Xu Z. Predictive criteria of severe cases in COVID-19 patients of early stage: a retrospective observational study. J Clin Lab Anal. 2020; e23562. 10.1002/jcla.23562 PMC7595922 32893398

[11] Jain V, Yuan JM (2020). Predictive symptoms and comorbidities for severe COVID-19 and intensive care unit admission: a systematic review and meta-analysis. Int J Public Health.

[12] Li Q, Zhang J, Ling Y, Li W, Zhang X, Lu H (2020). A simple algorithm helps early identification of SARS-CoV-2 infection patients with severe progression tendency. Infection.

[13] Liang M, He M, Tang J, He X, Liu Z, Feng S (2020). Novel risk scoring system for predicting acute respiratory distress syndrome among hospitalized patients with coronavirus disease 2019 in Wuhan, China. BMC Infect Dis.

[14] Liang W, Liang H, Ou L, Chen B, Chen A, Li C (2020). Development and validation of a clinical risk score to predict the occurrence of critical illness in hospitalized patients with COVID-19. JAMA Intern Med.

[15] Liu W, Tao ZW, Wang L, Yuan ML, Liu K, Zhou L (2020). Analysis of factors associated with disease outcomes in hospitalized patients with 2019 novel coronavirus disease. Chin Med J (Engl).

[16] Passamonti F, Cattaneo C, Arcaini L, Bruna R, Cavo M, Merli F (2020). Clinical characteristics and risk factors associated with COVID-19 severity in patients with haematological malignancies in Italy: a retrospective, multicentre, cohort study. Lancet Haematol..

[17] Schalekamp S, Huisman M, van Dijk RA, Boomsma MF, Freire Jorge PJ, de Boer WS (2020). Model-based prediction of critical illness in hospitalized patients with COVID-19. Radiology.

[18] Shang W, Dong J, Ren Y, Tian M, Li W, Hu J (2020). The value of clinical parameters in predicting the severity of COVID-19. J Med Virol.

[19] Tong X, Xu X, Lv G, Wang H, Cheng A, Wang D (2021). Clinical characteristics and outcome of influenza virus infection among adults hospitalized with severe COVID-19: a retrospective cohort study from Wuhan, China. BMC Infect Dis.

[20] Wang Z, Wang Z (2021). Identification of risk factors for in-hospital death of COVID - 19 pneumonia—lessions from the early outbreak. BMC Infect Dis.

[21] Wei YY, Wang RR, Zhang DW, Tu YH, Chen CS, Ji S (2020). Risk factors for severe COVID-19: evidence from 167 hospitalized patients in Anhui, China. J Infect.

[22] Yi P, Yang X, Ding C, Chen Y, Xu K, Ni Q (2020). Risk factors and clinical features of deterioration in COVID-19 patients in Zhejiang, China: a single-centre, retrospective study. BMC Infect Dis.

[23] Zeng Z, Wu C, Lin Z, Ye Y, Feng S, Fang Y (2021). Development and validation of a simple-to-use nomogram to predict the deterioration and survival of patients with COVID-19. BMC Infect Dis.

[24] Zhang J, Yu M, Tong S, Liu LY, Tang LV (2020). Predictive factors for disease progression in hospitalized patients with coronavirus disease 2019 in Wuhan, China. J Clin Virol.

[25] van Halem K, Bruyndonckx R, van der Hilst J, Cox J, Driesen P, Opsomer M (2020). Risk factors for mortality in hospitalized patients with COVID-19 at the start of the pandemic in Belgium: a retrospective cohort study. BMC Infect Dis.

[26] Huang D, Wang T, Chen Z, Yang H, Yao R, Liang Z (2020). A novel risk score to predict diagnosis with Coronavirus Disease 2019 (COVID-19) in suspected patients: a retrospective, multi-center, observational study. J Med Virol.

[27] Mao B, Liu Y, Chai YH, Jin XY, Lu HW, Yang JW (2020). Assessing risk factors for SARS-CoV-2 infection in patients presenting with symptoms in Shanghai, China: a multicentre, observational cohort study. Lancet Digit Health.

[28] Yu T, Cai S, Zheng Z, Cai X, Liu Y, Yin S (2020). Association between clinical manifestations and prognosis in patients with COVID-19. Clin Ther.

[29] Abate BB, Kassie AM, Kassaw MW, Aragie TG, Masresha SA (2020). Sex difference in coronavirus disease (COVID-19): a systematic review and meta-analysis. BMJ Open.

[30] Apra C, Caucheteux C, Mensch A, Mansour J, Bernaux M, Dechartes A, et al. Predictive usefulness of PCR testing in different patterns of Covid-19 symptomatology—analysis of a French cohort of 12,810 outpatients. medRxiv. 2020. 10.1038/s41598-021-99991-6 PMC8551264 34707198

[31] Menni C, Valdes AM, Freidin MB, Sudre CH, Nguyen LH, Drew DA (2020). Real-time tracking of self-reported symptoms to predict potential COVID-19. Nat Med.

[32] Alcoba-Florez J, Gil-Campesino H, de Artola GD, Gonzalez-Montelongo R, Valenzuela-Fernandez A, Ciuffreda L (2020). Sensitivity of different RT-qPCR solutions for SARS-CoV-2 detection. Int J Infect Dis.

[33] Fumagalli C, Rozzini R, Vannini M, Coccia F, Cesaroni G, Mazzeo F (2020). Clinical risk score to predict in-hospital mortality in COVID-19 patients: a retrospective cohort study. BMJ Open.

[34] Luo H, Liu S, Wang Y, Phillips-Howard PA, Ju S, Yang Y (2020). Age differences in clinical features and outcomes in patients with COVID-19, Jiangsu, China: a retrospective, multicentre cohort study. BMJ Open.

[35] Wang TY, Liu HL, Lin CY, Kuo FL, Yang PH, Yeh IJ (2020). Emerging success against the COVID-19 pandemic: hospital surge capacity in Taiwan. Ann Emerg Med.

[36] Zavascki AP, Gazzana MB, Bidart JP, P.S. F, A. G, Kawski CT, et al. Development of a predictive score for COVID-19 diagnosis based on demographics and symptoms in patients attended at a dedicated screening unit. medRxiv. 2020.

[37] Yang J, Zheng Y, Gou X, Pu K, Chen Z, Guo Q (2020). Prevalence of comorbidities and its effects in patients infected with SARS-CoV-2: a systematic review and meta-analysis. Int J Infect Dis.

[38] Romero-Gameros CA, Colin-Martinez T, Waizel-Haiat S, Vargas-Ortega G, Ferat-Osorio E, Guerrero-Paz JA (2021). Diagnostic accuracy of symptoms as a diagnostic tool for SARS-CoV 2 infection: a cross-sectional study in a cohort of 2,173 patients. BMC Infect Dis.

[39] Bertran Recasens B, Martinez-Llorens JM, Rodriguez-Sevilla JJ, Rubio MA (2020). Lack of dyspnea in patients with Covid-19: another neurological conundrum?. Eur J Neurol.

[40] Nouri-Vaskeh M, Sharifi A, Khalili N, Zand R, Sharifi A (2020). Dyspneic and non-dyspneic (silent) hypoxemia in COVID-19: possible neurological mechanism. Clin Neurol Neurosurg.

[41] Spechbach H, Jacquerioz F, Prendki V, Kaiser L, Smit M, Calmy A (2021). Network analysis of outpatients to identify predictive symptoms and combinations of symptoms associated with positive/negative SARS-CoV-2 nasopharyngeal swabs. Front Med (Lausanne).

[42] Tajlil A, Ghaffari S, Pourafkari L, Mashayekhi S, Roshanravan N (2020). Nicotine and smoking in the COVID-19 era. J Cardiovasc Thorac Res.

[43] Grant MC, Geoghegan L, Arbyn M, Mohammed Z, McGuinness L, Clarke EL (2020). The prevalence of symptoms in 24,410 adults infected by the novel coronavirus (SARS-CoV-2; COVID-19): a systematic review and meta-analysis of 148 studies from 9 countries. PLoS ONE.

[44] Menni C, Sudre CH, Steves CJ, Ourselin S, Spector TD (2020). Quantifying additional COVID-19 symptoms will save lives. Lancet.

[45] Spinato G, Fabbris C, Polesel J, Cazzador D, Borsetto D, Hopkins C (2020). Alterations in smell or taste in mildly symptomatic outpatients with SARS-CoV-2 infection. JAMA.

[46] Peyrony O, Marbeuf-Gueye C, Truong V, Giroud M, Riviere C, Khenissi K, et al. Accuracy of emergency department clinical findings for diagnosis of coronavirus disease 2019. Ann Emerg Med. 2020. 10.1016/j.annemergmed.2020.05.022 PMC7241345 32563600

[47] Abdi A, Jalilian M, Sarbarzeh PA, Vlaisavljevic Z (2020). Diabetes and COVID-19: A systematic review on the current evidences. Diabetes Res Clin Pract.

[48] Guan WJ, Ni ZY, Hu Y, Liang WH, Ou CQ, He JX (2020). clinical characteristics of coronavirus disease 2019 in China. N Engl J Med.

[49] Guo FR (2020). Active smoking is associated with severity of coronavirus disease 2019 (COVID-19): an update of a meta-analysis. Tob Induc Dis.

[50] Reddy RK, Charles WN, Sklavounos A, Dutt A, Seed PT, Khajuria A (2020). The effect of smoking on COVID-19 severity: a systematic review and meta-analysis. J Med Virol.

[51] Vardavas CI, Nikitara K (2020). COVID-19 and smoking: a systematic review of the evidence. Tob Induc Dis.

[52] Adrish M, Chilimuri S, Mantri N, Sun H, Zahid M, Gongati S (2020). Association of smoking status with outcomes in hospitalised patients with COVID-19. BMJ Open Respir Res.

[53] Fink N, Rueckel J, Kaestle S, Schwarze V, Gresser E, Hoppe B (2021). Evaluation of patients with respiratory infections during the first pandemic wave in Germany: characteristics of COVID-19 versus non-COVID-19 patients. BMC Infect Dis.

[54] Lien WC, Wu JL, Tseng WP, Chow-In Ko P, Chen SY, Tsai MS (2020). Fight COVID-19 beyond the borders: emergency department patient diversion in Taiwan. Ann Emerg Med.

