# Supplementary material for: A new screening tool for SARS-CoV-2 infection based on self-reported patient clinical characteristics: the COV19-ID score
Source: BMC Infect Dis. 2022 Feb 24;22:187. doi: 10.1186/s12879-022-07164-1 (PMC8867452; doi:10.1186/s12879-022-07164-1)
Supplement: Supplementary file 1 — Additional file 1: Predicted probabilities of SARS-COV-2 infection compared to RT-PCR test results. [file 12879_2022_7164_MOESM1_ESM.docx]

**

**Additional file 1:** Predicted probabilities of SARS-COV-2 infection compared to RT-PCR test results
