# Supplementary material for: A new screening tool for SARS-CoV-2 infection based on self-reported patient clinical characteristics: the COV19-ID score
Source: BMC Infect Dis. 2022 Feb 24;22:187. doi: 10.1186/s12879-022-07164-1 (PMC8867452; doi:10.1186/s12879-022-07164-1)
Supplement: Supplementary file 2 — Additional file 2: Model performance on the test dataset using three different thresholds to maximize either sensitivity, specificity, or both. [file 12879_2022_7164_MOESM2_ESM.docx]

| **Additional file 2: Model performance on the test dataset using three different thresholds to maximize either sensitivity, specificity, or both** | | | | | | | | | |
| --- | --- | --- | --- | --- | --- | --- | --- | --- | --- |
|  |  |  |  |  |  |  |  |  |  |
|  |  | Maximize sensitivity (Threshold 8.5 points) | |  | Maximize sensitivity and specificity  (Threshold 14 points) | |  | Maximize specificity (Threshold 25 points) | |
|  |  | Actual | Bootstrap (95% CI) |  | Actual | Bootstrap (95% CI) |  | Actual | Bootstrap (95% CI) |
|  |  |  |  |  |  |  |  |  |  |
|  |  |  |  |  |  |  |  |  |  |
|  | True positive (TP) | 378 |  |  | 345 |  |  | 243 |  |
|  | True negative (TN) | 745 |  |  | 1001 |  |  | 1217 |  |
|  | False positive (FP) | 641 |  |  | 385 |  |  | 169 |  |
|  | False negative (FN) | 51 |  |  | 84 |  |  | 186 |  |
|  | Accuracy | 61.9% | (61.8% – 62.0%) |  | 74.2% | (74.1% – 74.3%) |  | 80.4% | (80.4% – 80.5%) |
|  | Sensitivity | 88.1% | (88.1% – 88.3%) |  | 80.4% | (80.4% – 80.6%) |  | 56.6% | (56.6% – 56.9%) |
|  | Specificity | 53.8% | (53.7% – 53.9%) |  | 72.2% | (72.2% – 72.3%) |  | 87.8% | (87.7% – 87.8%) |
|  | Positive Predictive Value (PPV) | 37.1% | (37.0% – 37.2%) |  | 47.3% | (47.2% – 47.4%) |  | 59.0% | (58.7% – 59.0%) |
|  | Negative Predictive Value (NPV) | 93.6% | (93.6% – 93.7%) |  | 92.3% | (92.3% – 92.4%) |  | 86.7% | (86.7% – 86.8%) |
|  | Positive likelihood ratio (LR+) | 1.90 | (1.90 – 1.91) |  | 2.90 | (2.90 – 2.91) |  | 4.65 | (4.64 – 4.69) |
|  | Negative likelihood ratio (LR-) | 0.22 | (0.22 – 0.22) |  | 0.27 | (0.27 – 0.27) |  | 0.49 | (0.49 – 0.50) |
|  | F1 score | 0.52 | (0.51 – 0.53) |  | 0.60 | (0.59 – 0.60) |  | 0.58 | (0.58 – 0.59) |
|  | Mathews correlation coefficient (MCC) | 0.36 | (0.36 – 0.36) |  | 0.46 | (0.46 – 0.46) |  | 0.45 | (0.45 – 0.45) |
|  |  |  |  |  |  |  |  |  |  |
|  |  |  |  |  |  |  |  |  |  |
